# Analysis of the condition of thin-walled composite structures based on the results of experiments conducted at variable temperature by acoustic emission

**DOI:** 10.1038/s41598-026-40593-5

**Published:** 2026-02-22

**Authors:** Tomasz Kopecki, Lukasz Swiech, Patryk Rozylo, Pawel Wysmulski, Anna Falkowska, Adam Tomczyk

**Affiliations:** 1 Department of Aerospace Engineering, Rzeszow University of Technology, Powstancow Warszawy 8, Rzeszow, Poland; 2 Department of Machine Design and Mechatronics, Lublin University of Technology, Nadbystrzycka 36, Lublin, Poland; 3 Department of Mechanics and Applied Computer Science, Bialystok University of Technology, Wiejska 45C, Bialystok, Poland

**Keywords:** Temperature impact, Thin-walled composite structures, Post-buckling, Load-carrying capacity, Engineering, Materials science, Physics

## Abstract

This paper presents considerations for interpreting acoustic emission results in combination with equilibrium paths obtained from experiments conducted using a testing machine and a digital image correlation system. The experiments were performed on thin-walled carbon-epoxy composite profiles. Two types of samples with different shapes were tested at various temperatures. The samples had identical lay-up configurations. The primary objective of the research was to perform a quantitative and qualitative assessment of composite material damage using independent experimental testing techniques, including digital image correlation (DIC) and acoustic emission. An additional aspect was to assess the impact of temperature on the stability and load-bearing capacity of thin-walled composite structures under variable thermal conditions (negative and positive temperatures). The paper used experimental, non-destructive testing methods to evaluate how two types of composite structures behave under different conditions and under simultaneous temperature variations.

## Introduction

Thin-walled composite structures are load-bearing systems increasingly used in aerospace and automotive engineering. These components typically have shell forms, but most aircraft load-bearing members are in the form of bar structures, such as fuselage stringers, wing spars, frames, and ribs. These structures usually have thin-walled profiles with different cross-sections and fibre orientations.

As aircraft components, these structures are exposed to a wide range of temperatures, from very low temperatures characteristic of high cruising altitudes to relatively high temperatures resulting from heating during stops in sunny locations in hot climate zones. During the run-up and take-off phases following the standstill phase, the structure is forced to operate over the full load range before reaching a state of significant cooling. The mechanical properties of fibrous composite materials are sensitive to temperature variations. Composite members of load-bearing structures should be tested under laboratory thermal conditions that reflect the operating conditions.

Bar structures are prone to compression-induced buckling, which negatively impacts their structural integrity^1–5^. Although global buckling is considered structural failure under established doctrine, postbuckling behavior arising from local elastic effects in thin-walled structures may be acceptable, provided relevant assumptions regarding the nature and magnitude of postbuckling are satisfied^6–9^. Therefore, it is necessary to investigate in detail the buckling and postbuckling processes in a given thin-walled profile through experiments^3,10,11^.

During the operation of aircraft or vehicles containing thin-walled composite structures as load-bearing components, it is very difficult - if not impossible - to carry out ongoing measurements of their geometrical parameters, to detect symptoms of potential composite damage^12–14^. It is therefore necessary to use tools that measure physical quantities accompanying damage formation^15–17^. Acoustic signal emission analysis is one such tool that is relatively easy to use and fairly inexpensive to apply under operational conditions. Therefore, experiments conducted on thin-walled composite profiles must include this type of analysis^18,19^.

Local buckling effects can initiate structural delamination, which, however, need not disrupt the system’s representative equilibrium paths^20,21^. A comparative analysis of equilibrium paths and acoustic signals can serve as a tool for estimating the risk of structural damage under given operating conditions.

The scope of previous research on this type of load-bearing system is quite broad. Most studies have focused on buckling in thin-walled elastic profiles, with particular emphasis on methods for determining critical loads^22–24^. Problems related to the load-bearing capacity of thin-walled composite profiles were also studied^25,26^. Many studies investigated the effect of the number of matrix fabric layers and their fibre orientations on the mechanical properties of composite profiles, including the mechanisms of buckling and failure processes^6,14^. Several studies also involved conducting experiments using acoustic signal analysis.

This study does not focus on the problems of determining critical loads, since most of the effects induced by composite profile delamination occur in the postbuckling load range. The primary objective of this study was to identify relationships between physical phenomena observed during experiments and to develop conclusions and recommendations for predicting damage in load-bearing structures during operation. Methods such as digital image correlation (DIC) and acoustic emission were used. These methods were successfully applied to analyse the compression of very long, thick composite tubes^27^. Representative equilibrium paths describing the relationship between profile shortening and compressive load were also determined^28^.

The novelty of the presented approach lies in the presentation of research results on thin-walled composite profiles with Ω-type (Type 1) and Z-type (Type 2) cross-sections, composed of 10 individual layers with different fibre orientation sequences. The behaviour of such elements in variable environments, especially at lowered temperatures, is particularly interesting. Given the limited results of previous experimental studies, this is an extremely interesting aspect, especially in the context of using profiles for aerospace components. This work aims to use three different research methods and identify common relationships among the results obtained, especially under variable (both reduced and increased) temperature conditions. This will enable the prediction of cracking in thin-walled composite profile structural elements over a wide range of temperatures.

### The object and scope of the study

**Fig. 1:**
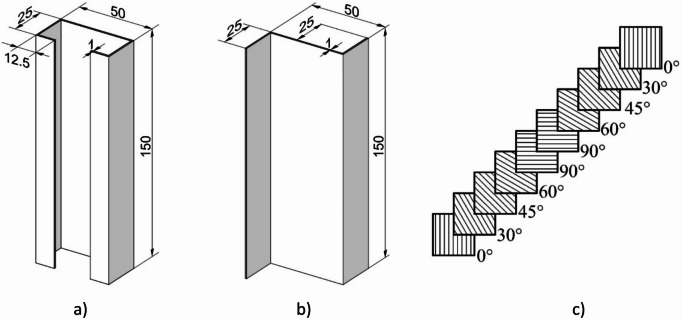
Geometrical parameters of test samples: **(a)** type I, **(b)** type II, **(c)** schematic representation of 10 plies according to designation (0/30/45/60/90/90/60/45/30/0).

The study was conducted on compressed thin-walled composite profiles, whose geometrical parameters are shown in Fig. 1. Test samples were fabricated from a carbon/epoxy composite material by autoclave curing. The fabrication process was highly accurate and repeatable. For this purpose, a unidirectional prepreg manufactured by Wit-Composites (Poland) was used.

Mechanical properties of the material were determined in compliance with the following standards: PN-EN ISO 527-5 (ASTM D 3039) – for tensile testing, PN-EN ISO 14,129 (ASTM D 3518) – for shear stress determination, and PN-EN ISO 14,126 (ASTM D 3410) – for compressive testing, which are described in detail in^29,30^. A comparison of the mechanical properties of the material is given in Table 1.

The composite structure of each model consisted of 10 plies, symmetric to the mid-plane (Fig. 1c). All tested profiles had the same layup of (0/30/45/60/90/90/60/45/30/0)^31^. Where the 0 direction is in line with the longitudinal axis of the sample.

**Table 1:** Mechanical properties of the tested material for different temperatures.

Temp.	E1	E2	ν	G	F_1t	F_2t	F_12shr	F_1c	F_2c
[ºC]	[GPa]	[GPa]	-	[GPa]	[MPa]	[MPa]	[MPa]	[MPa]	[MPa]
20	118.3	8.3	0.32	4.6	1781.4	49.1	137.5	707.7	115.5
40	121.0	8.0	0.32	4.3	1511.3	50.7	118.2	706.9	113.2
60	124.3	7.3	0.34	3.5	1387.3	49.0	96.1	484.8	77.9
80	128.0	5.5	0.39	1.9	1281.9	38.5	80.7	358.3	65.2

**Fig. 2:**
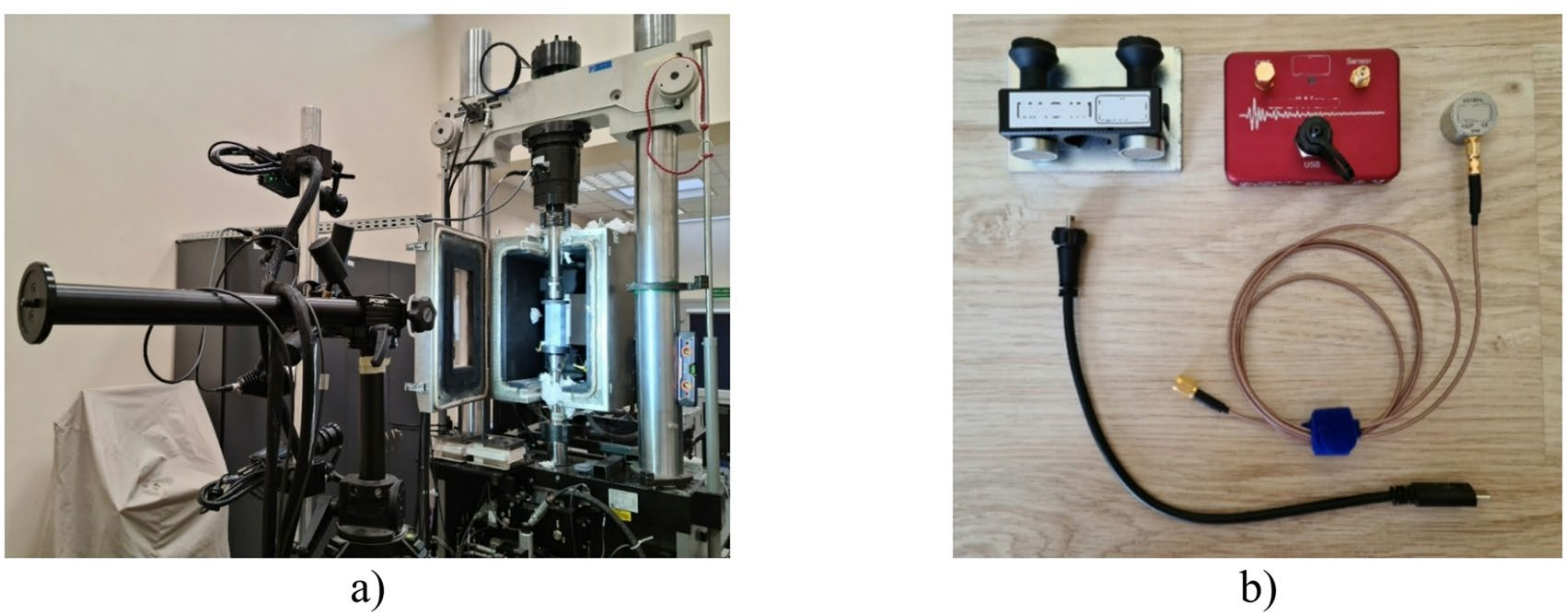
Experimental setup **(a)** and acoustic emission measuring equipment **(b)**.

Experiments were conducted on an MTS 809 testing machine having a load range of up to 100kN and equipped with an insulated thermal chamber allowing liquid nitrogen cooling (Fig. 2a). The experiments could hence be conducted both at the reference temperature (20 °C) and at the temperatures of −20 °C, 40 °C, 60 °C, and 80 °C^32^. The crosshead speed was maintained at 1 mm/min during all stages of the experiments.

An optical scanner based on the GOM Aramis 5 M DIC method was used to ensure accurate measurement of the deflection of selected points in each model^33,34^. The employed measurement method made it possible to determine representative equilibrium paths describing the relationship between shortening and load, as well as to obtain insight into the nature and magnitude of profile wall buckling^35,36^.

Acoustic emission measurements were carried out using a single-channel SpotWave 201 system from Vallen Systeme with a 100dB-range piezoelectric sensor, VS150-L (Fig. 2b). This sensor is used for making measurements on thin-walled structures and aluminium profiles. The sensor was attached directly to the test sample.

### Experiments

**Fig. 3:**
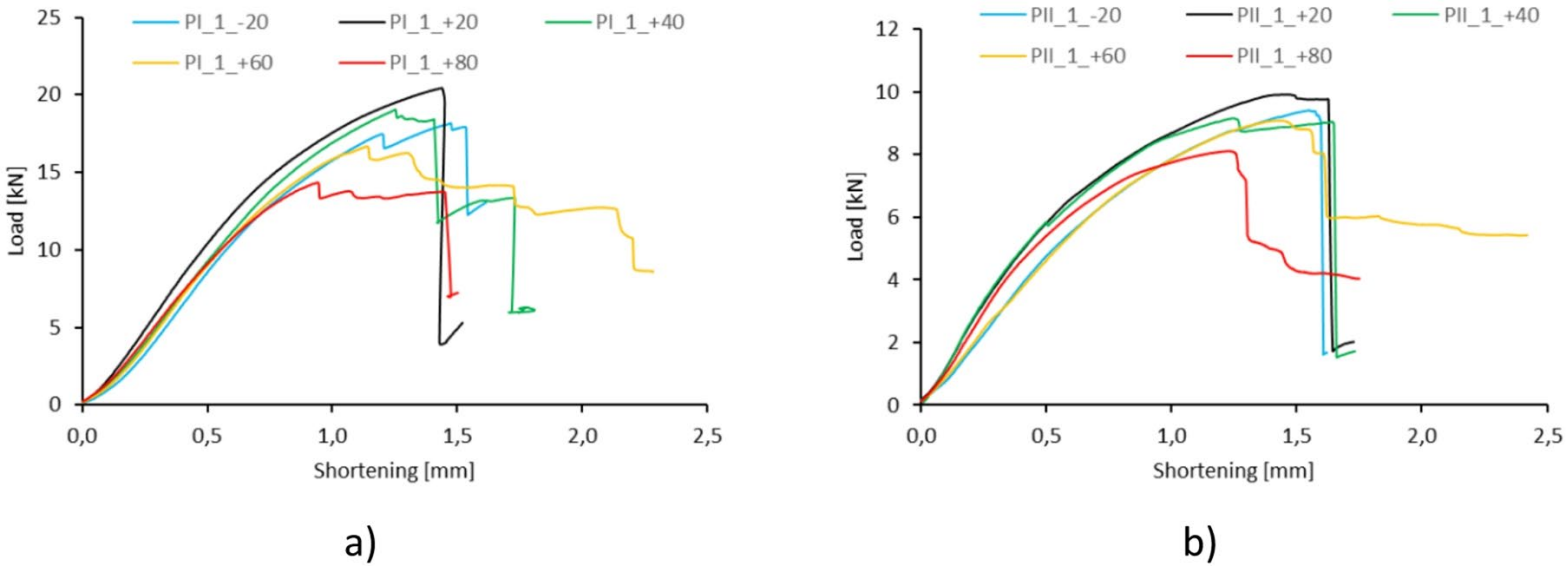
Representative equilibrium paths for specimen type I **(a)** and type II **(b)**.

The experiments made it possible to determine representative equilibrium paths for all temperatures. As previously mentioned, these paths describe the relationship between absolute shortening and compressive load (Fig. 3).

**Fig. 4:**
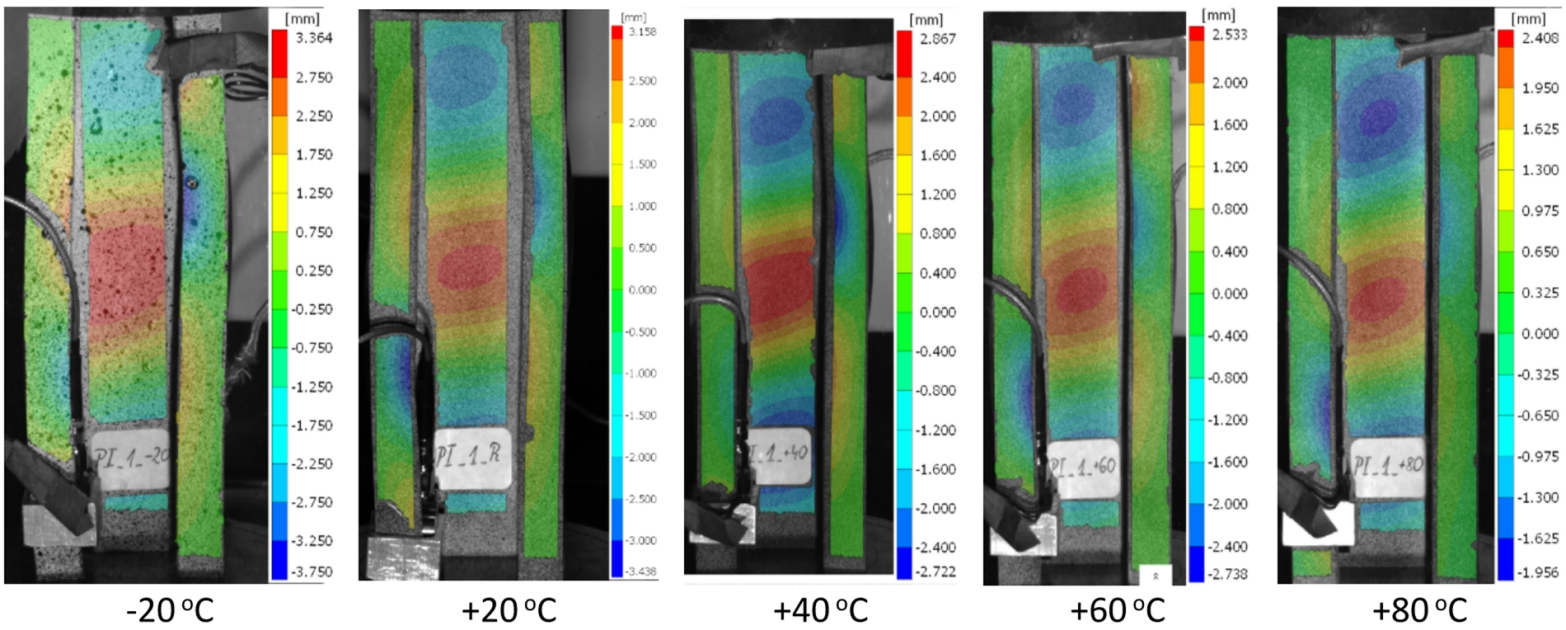
Magnitude of deformations for the maximum load for type I samples. DIC result.

The use of the DIC scanner enabled the determination of deformation fields on the surfaces of type I samples at different temperatures. Figure 4 shows the magnitude of deformation at the maximum load for the samples. It should be noted that in each case considered, the loss of stability manifested itself as three half-waves of deflection of the profile web along its height. This pattern persisted throughout the experiments.

**Fig. 5:**
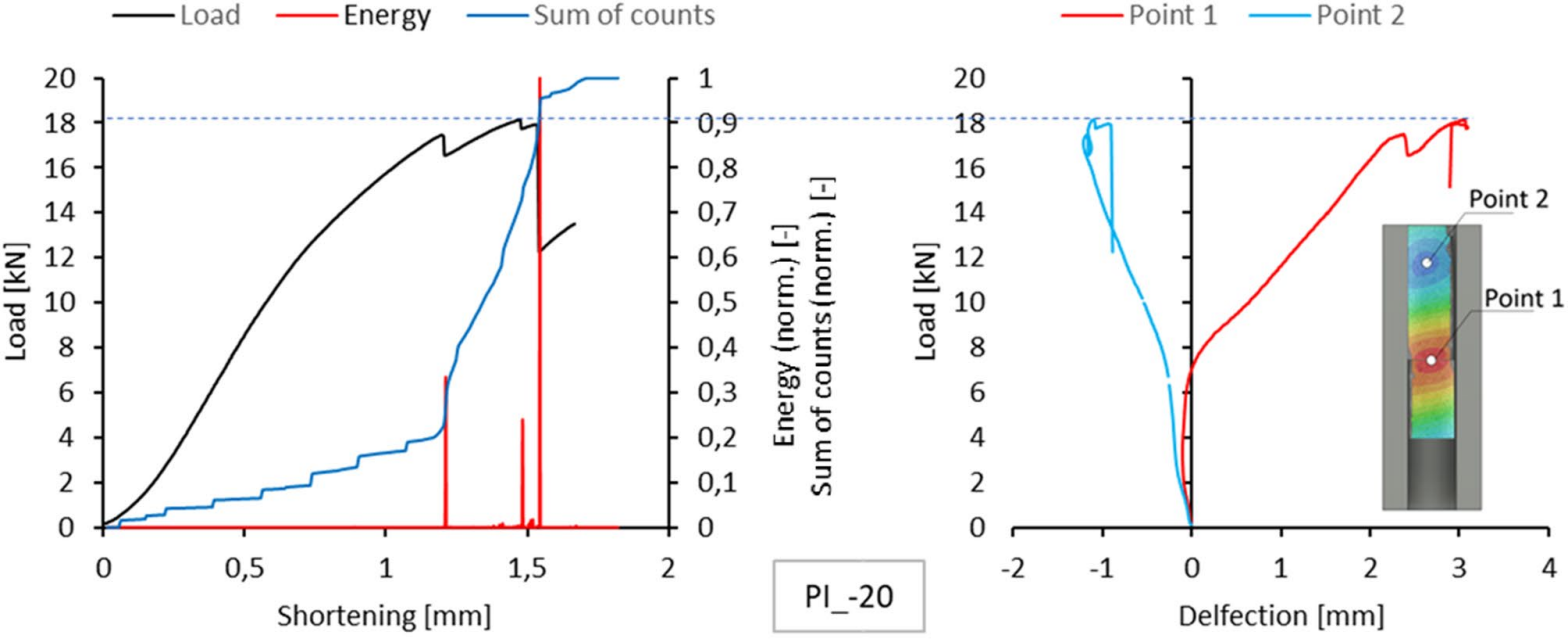
Comparison of acoustic emission results and local equilibrium paths for type I specimen tested in the temperature of −20 °C.

**Fig. 6:**
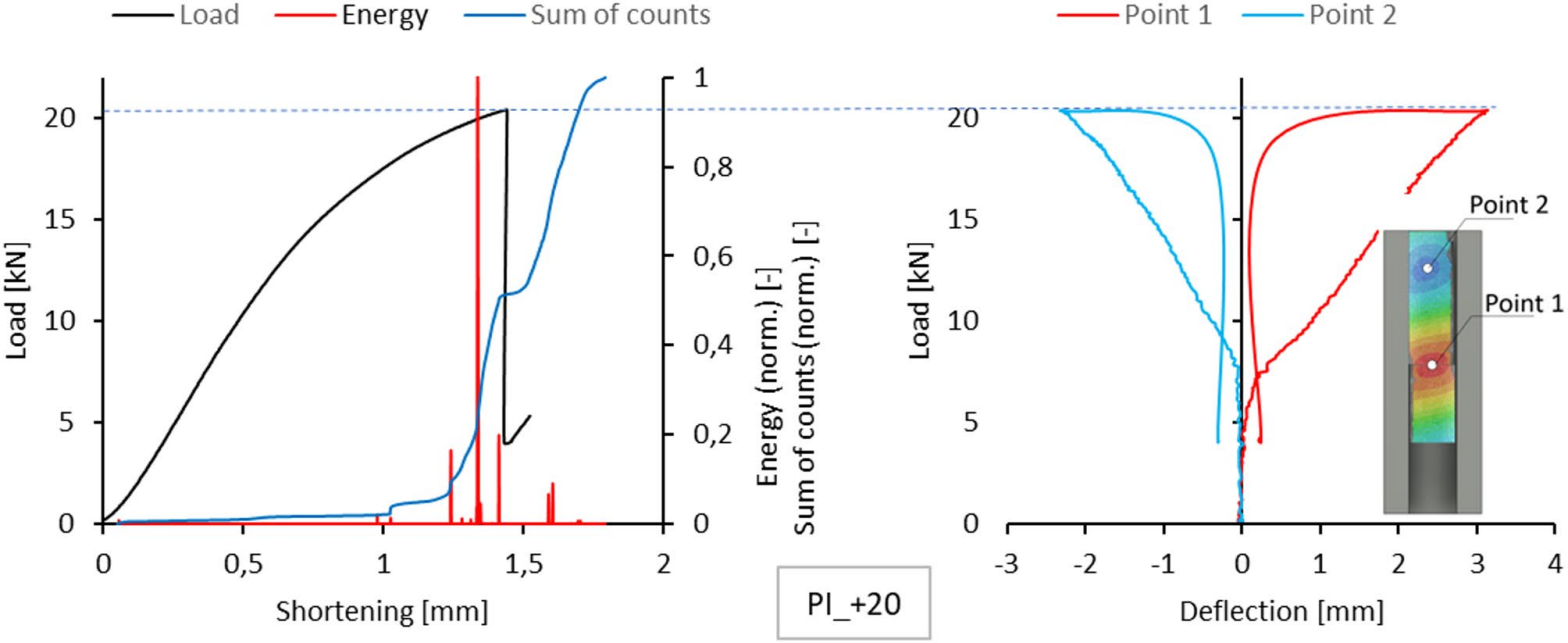
Comparison of acoustic emission results and local equilibrium paths for type I specimen tested in the temperature of + 20 °C.

**Fig. 7:**
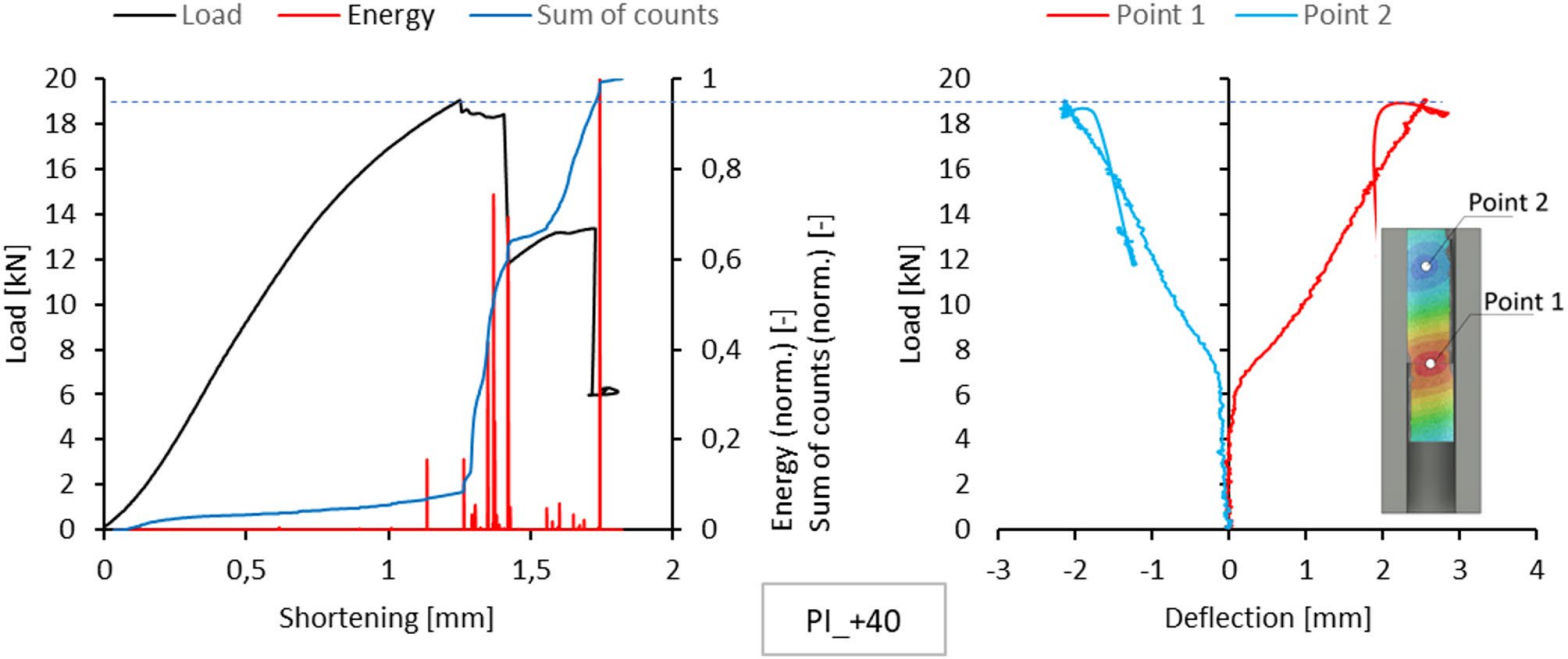
Comparison of acoustic emission results and local equilibrium paths for type I specimen tested in the temperature of + 40 °C.

**Fig. 8:**
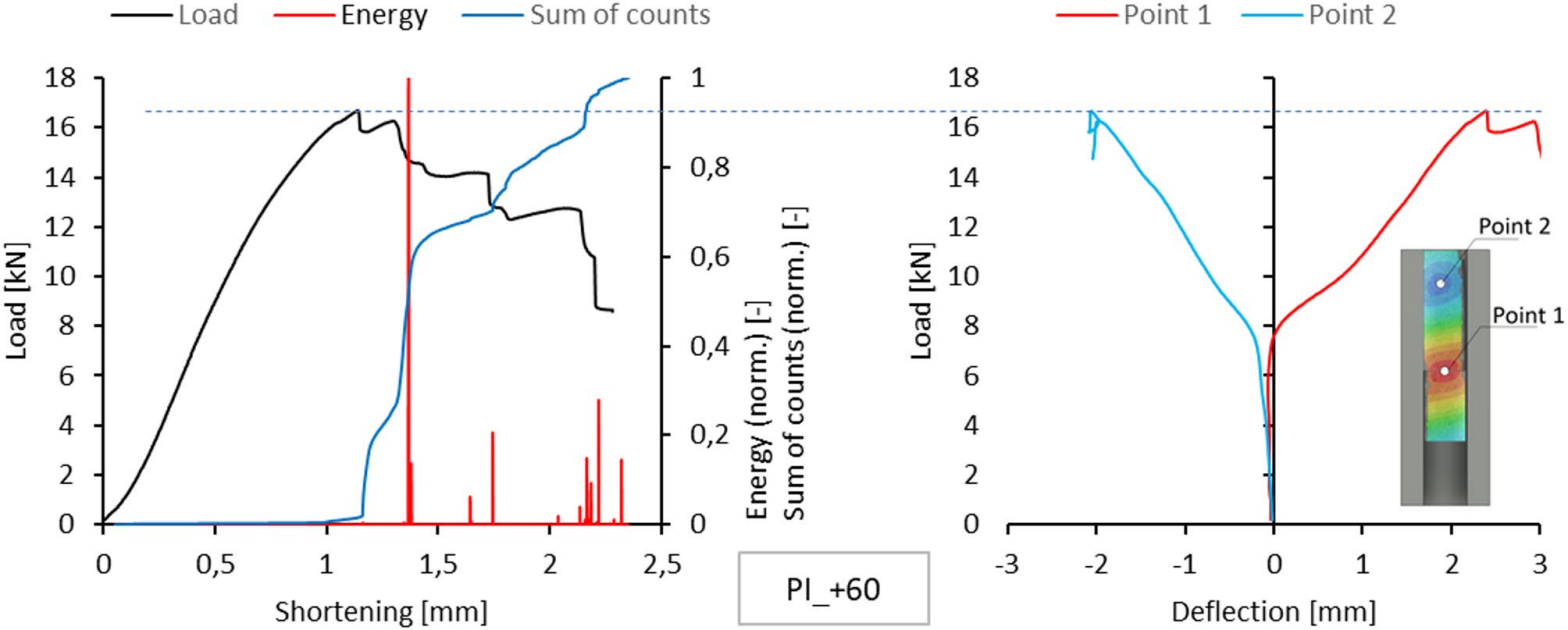
Comparison of acoustic emission results and local equilibrium paths for type I specimen tested in the temperature of + 60 °C.

**Fig. 9:**
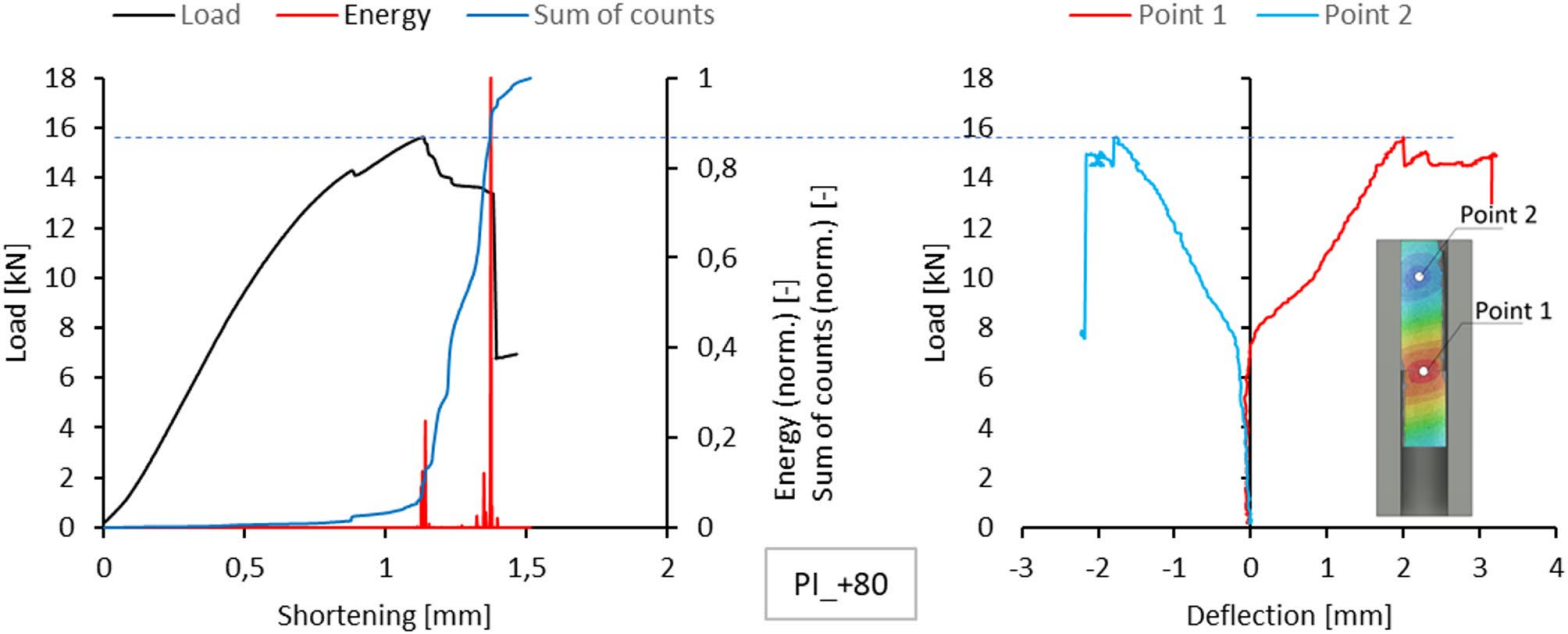
Comparison of acoustic emission results and local equilibrium paths for type I specimen tested in the temperature of + 80 °C.

Acoustic emission measurements provided lists of characteristics for selected temperatures for each sample. This included plotting the energy, amplitude, number and sum of signal counts as a function of load. These charts are attached as an appendix to the work. However, the text of the paper is limited to presenting the comparison of normalized energy curves and sums of counts with the compression graph of the samples, which can be treated as the global equilibrium path of the tested structure. This simplification is justified because the evaluation of acoustic emission test results relies on analysing the changes, not specific measured values. The graphs on the left side of Figs. 5, 6, 7, 8 and 9 present the values ​​described above.

Since a local loss of stability of the profile web was observed during the tests, using the results of measurements with the DIC scanner, it was possible to plot equilibrium paths for characteristic points on the surface of the tested samples. The representative points were selected as the point near the middle of the profile height, where the greatest positive deflection was observed (Point 1), and the point of maximum deflection in the opposite direction in the part closer to the upper edge of the profile (Point 2). The deflection graphs of these points (deformation in the direction perpendicular to the web) are shown on the right side of Figs. 5, 6, 7, 8 and 9.

Based on the research conducted, it was observed that an increase in the energy signal at a local point (usually associated with a sudden increase in the sum of counts) corresponds to the initiation or evolution of composite damage. Typically, local increases in acoustic signals correspond to a change in the experimental load-shortening curve (noticeable decreases in load increase). Most often, local increases in acoustic signals occur sometime before the loss of load-bearing capacity (maximum recorded load), indicating the initiation phase of damage before failure.

**Fig. 10:**
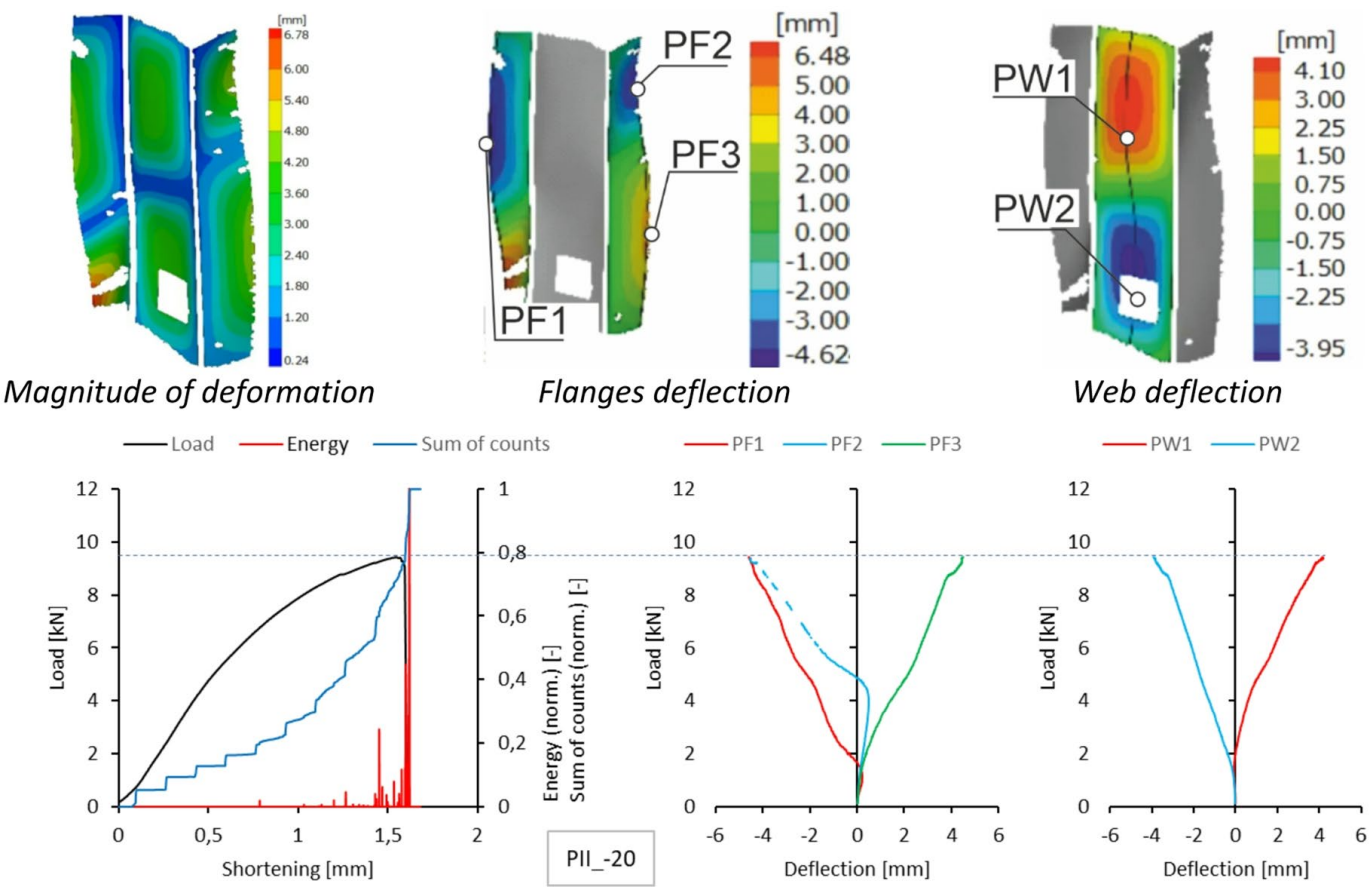
Deformation fields from DIC, comparison of acoustic emission results and local equilibrium paths for type II specimen tested in the temperature of −20 °C.

**Fig. 11:**
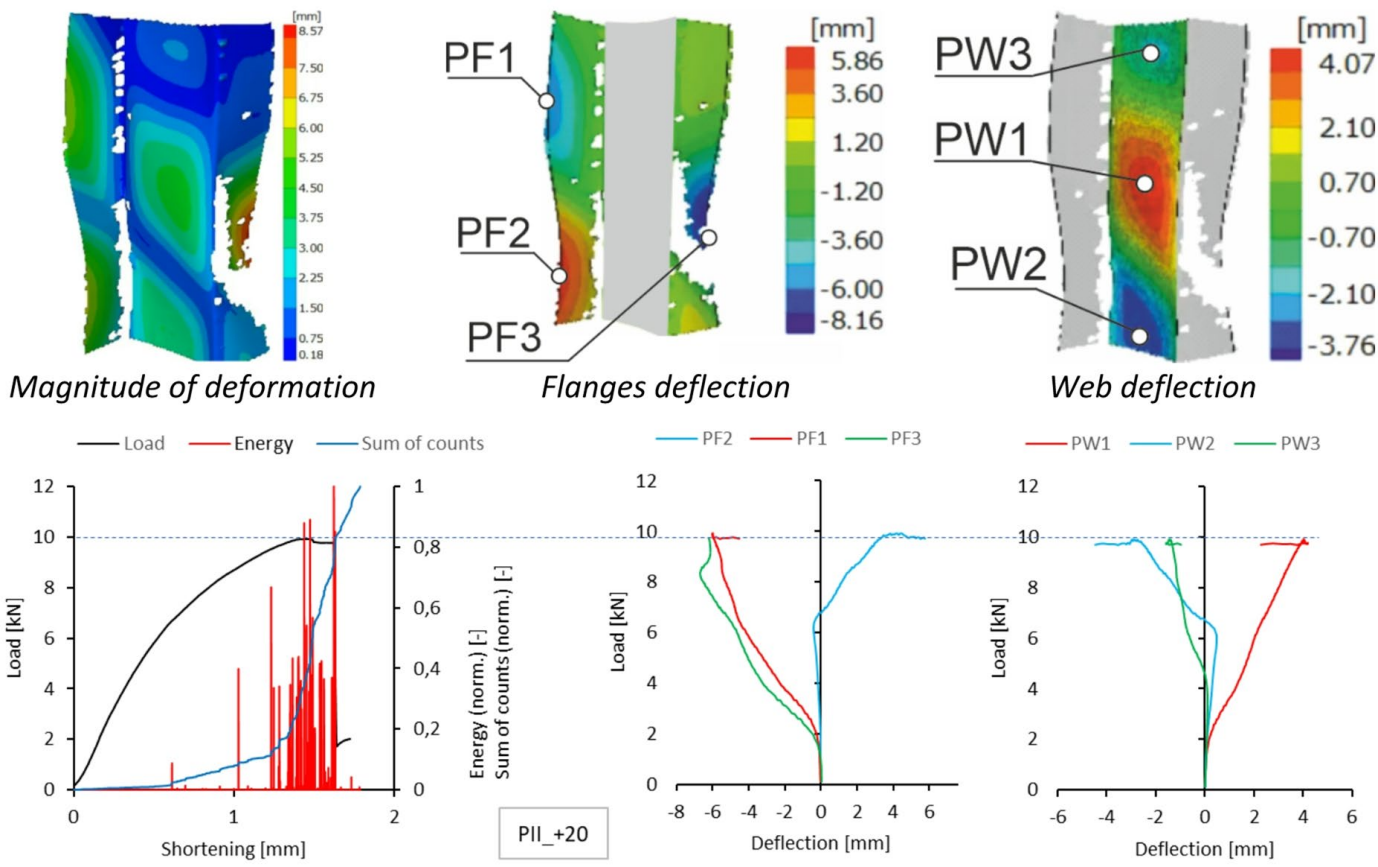
Deformation fields from DIC, comparison of acoustic emission results and local equilibrium paths for type II specimen tested in the temperature of + 20 °C.

**Fig. 12:**
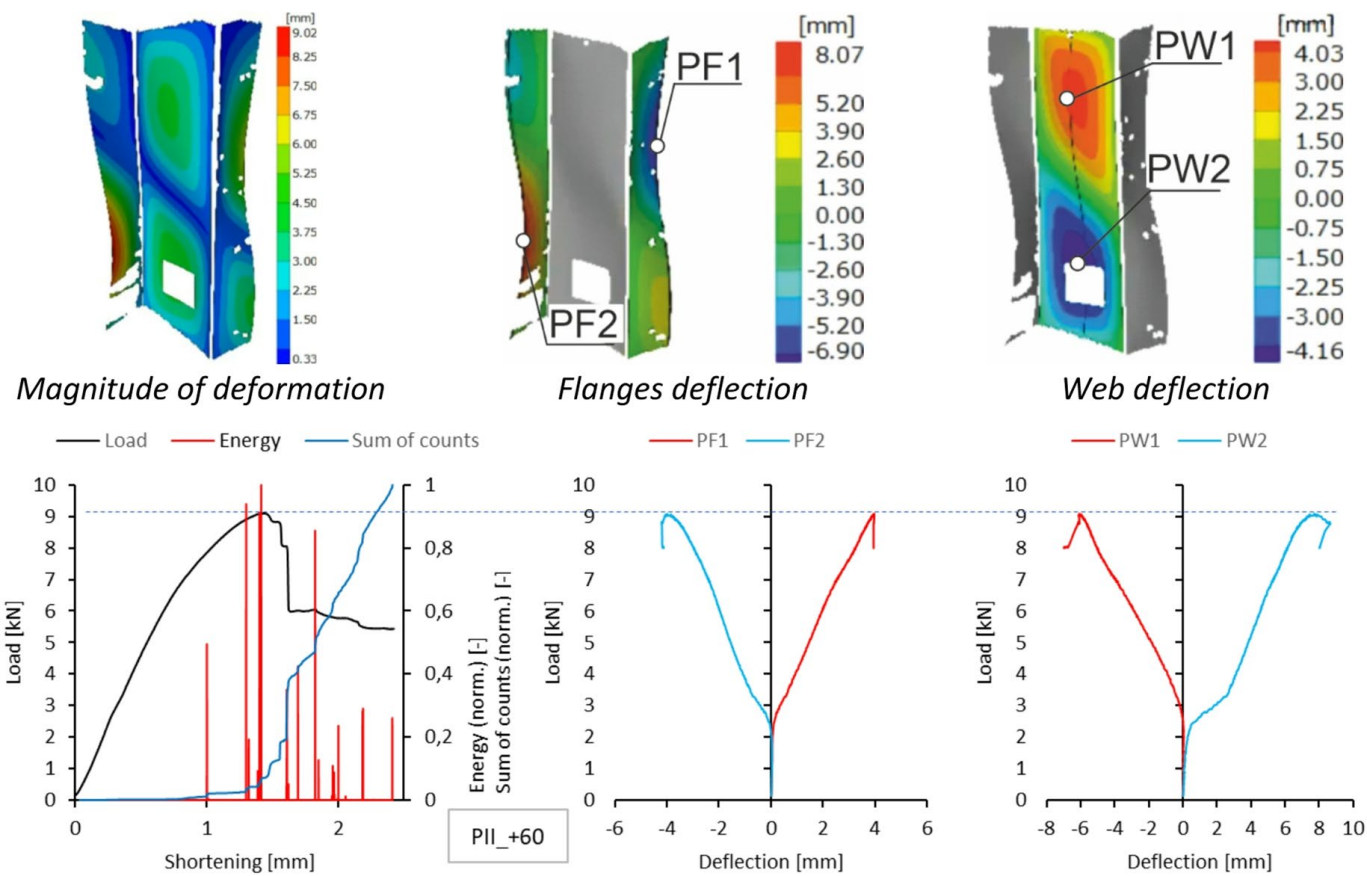
Deformation fields from DIC, comparison of acoustic emission results and local equilibrium paths for type II specimen tested in the temperature of + 60 °C.

**Fig. 13:**
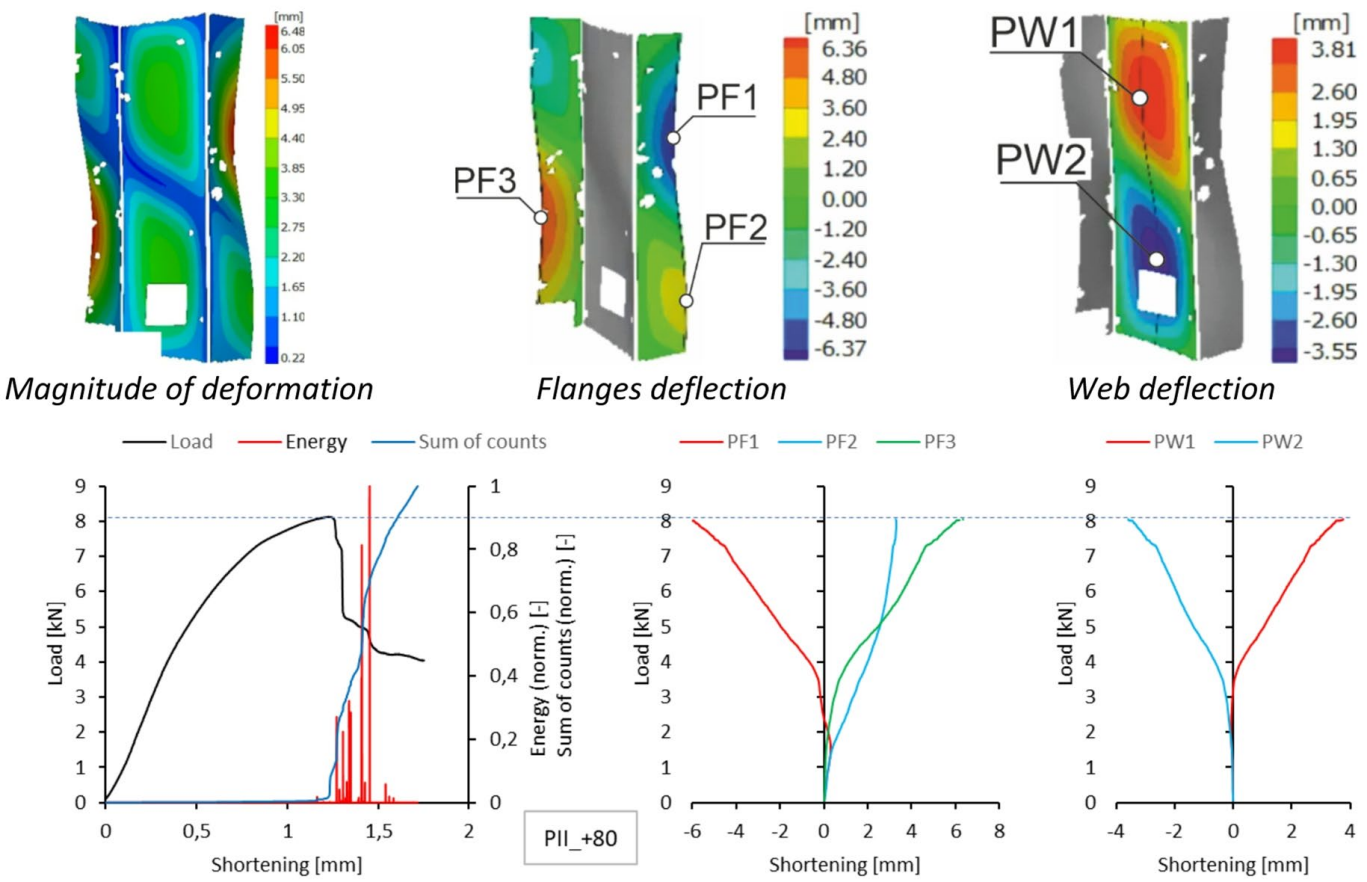
Deformation fields from DIC, comparison of acoustic emission results and local equilibrium paths for type II specimen tested in the temperature of + 80 °C.

A similar summary of results is provided for type II samples. DIC scanner results, including the magnitude of deformation at the maximum load level and the deflection of the walls and flanges at experimental temperatures, are presented in Figs. 10, 11, 12 and 13. As shown, the form of stability loss in type II samples is more complex. Deflection is understood as deformation normal to the surface of the considered part of the profile.

At all test temperatures except the reference temperature, profile web buckling was observed as two deflection half-waves. In the test at the reference temperature, the loss of stability manifested as three deflections, with the deflection in the upper part of the profile web being smaller. In each case, the profile flanges assumed the shape of two deflection half-waves. Due to minor complications during the experimental research, no characteristics for sample type II at + 40 °C were obtained because recording the experimental data was problematic.

**Fig. 14:**
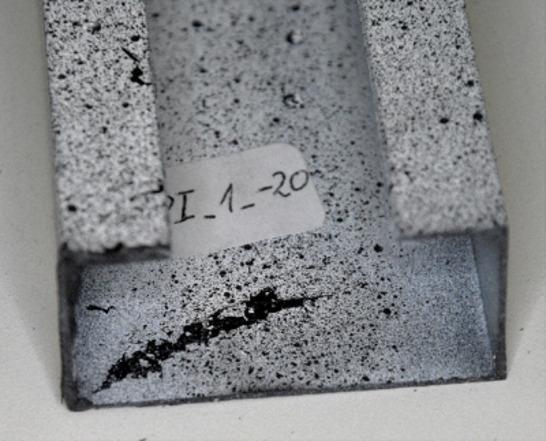
Failure of a type I sample at −20 °C.

The number and sum of signal counts obtained from the experiment at −20 °C are distorted by acoustic effects generated by the cooling apparatus, which systematically exceed the threshold. However, the acoustic energy signals for both sample types have noticeable features in common. A significant increase in the signal energy as the load approaches its maximum value suggests rapid damage to the composite structure. In the type I sample, this effect is accompanied by a clear drop in the load value, evident in the equilibrium path. The subsequent increase in acoustic signal energy, slightly lower than previously, indicates the progression of damage and is directly preceded by structural failure. A photograph of the failure area in the sample is shown in Fig. 14. For a sample of type II, the damage progression is less rapid compared to that observed for type I, and the signal energy increments are much lower. This is due to the sample’s higher susceptibility owing to its cross-sectional shape. The optical scanning results obtained for both sample types show discrepancies between the deflections of the analysed points. To give an example, for the type I sample, the load value corresponding to the first large increase in the signal energy at Point 1 caused a sudden increase in the deflection of this point first, and then a load decrease corresponding to a decline in the representative equilibrium path.

For the type II sample, the first significant increase in the acoustic signal energy was accompanied by a rapid increment in the deflection of Points 2 and 3.

**Fig. 15:**
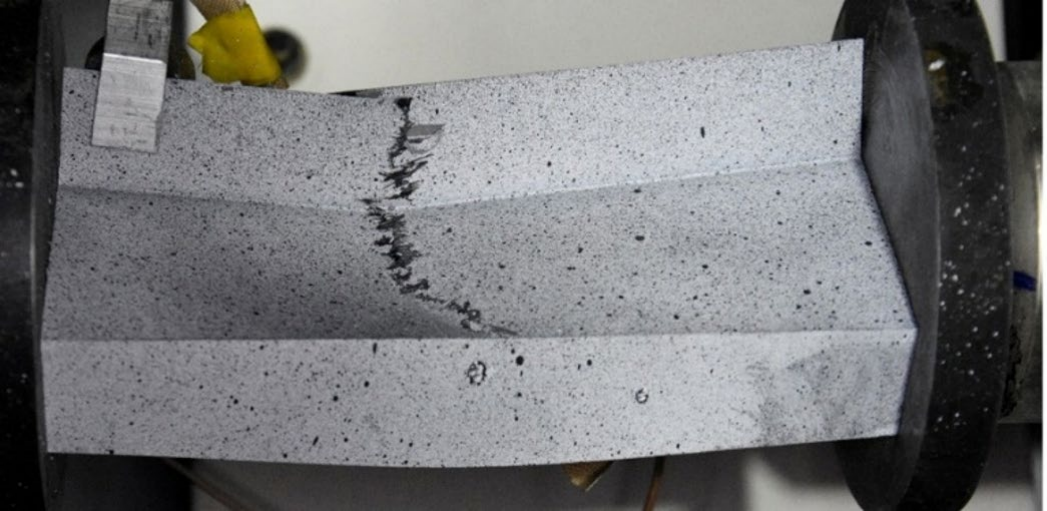
Failure of a type II sample at −20 °C.

It should be noted that for both types of samples, no significant increase in the energy signal was observed at earlier phases of the load increment, suggesting a complete absence of damage in the composite structure. Thus, the first of these increments indicates that a further load increase will lead to imminent failure (Fig. 15).

A slightly different mechanism of damage progression was observed in the experiment conducted at the reference temperature of + 20 °C. Given the absence of significant interference with the acoustic signals, the results for the number of counts and the sum of signal counts are reliable for damage progression assessment.

**Fig. 16:**
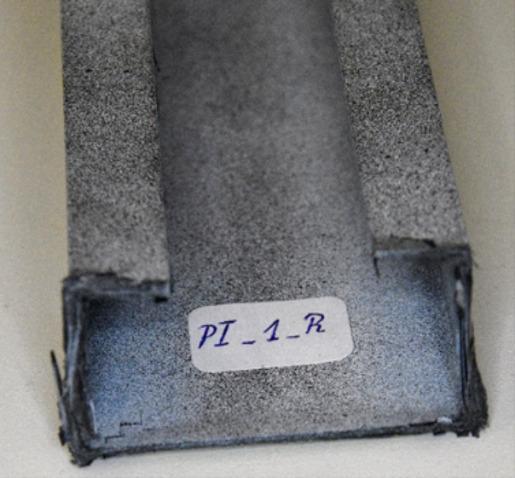
Failure mode of type I sample at reference temperature.

For type I samples, a significant increase in counts was observed at a shortening value of approximately 1 mm. This is evident in the plot showing the sum of counts as a function of load. With a further increase in load, a significant increase in signal energy is observed, with values corresponding to a large increase in the gradient of the sum of counts until structural failure. The optical scanning results do not reveal any rapid increase in the deflection of the analysed points on the sample. The loss of structural stability occurred at a relatively low load and was not related to failure. The failure of the composite structure took place in the immediate vicinity of the testing machine plates, i.e., near the end sections of the composite column (Fig. 16).

For type II samples, a clear increase in the gradient of the sum of counts occurred at a load value of about 7 kN, for which the optical scanning results revealed a sharp increase in the deflection of Point 2, which can be attributed to bifurcation and postbuckling mode transition. In the acoustic signal energy plot, one can see the first visible increase in the deflection for this load value. Although the bifurcation itself may be a source of acoustic emission, the observed systematic increase in the number of counts proves that the local delamination developing inside the composite structure was the primary source of acoustic emission.

It can therefore be concluded that the changes in the gradients of the sum of counts for both types of samples make it possible to determine the damage-initiating load for the composite structure.

**Fig. 17:**
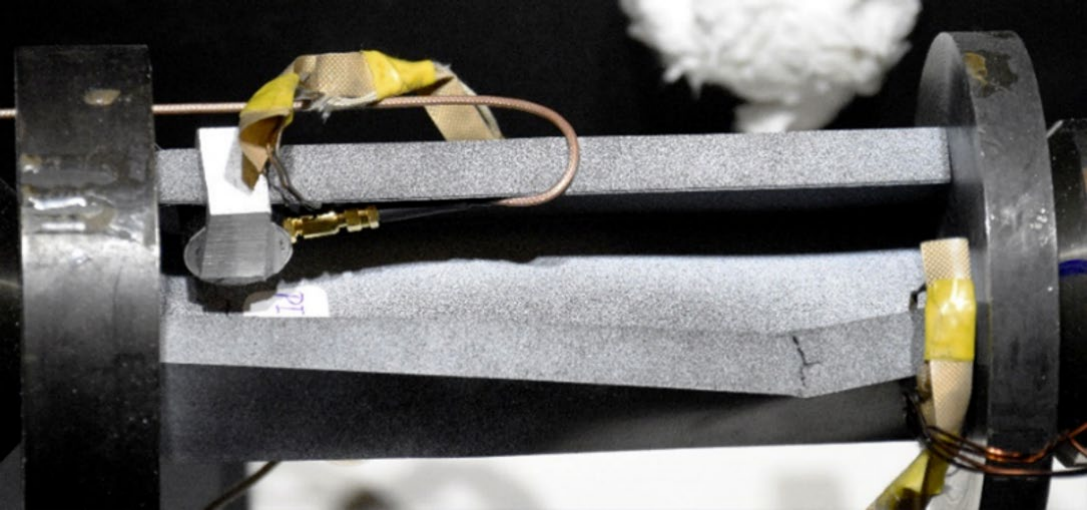
Failure of type I sample at 40 °C.

At + 40 °C, the samples failed after increases in the acoustic signal energy, corresponding to a decline in the representative equilibrium paths. The onset of these increments occurred at the maximum load, resulting in approximately 1.3 mm of shortening in both sample types. This moment marks the initiation of composite failure. Further on in the experiment, delamination occurred in stages. It can be assumed that the delamination was caused by interactions between the reinforcement and the composite matrix that differed from those observed in previous cases. Like in the reference temperature case, the initiation of intensive damage processes in the samples was signalled by a sudden increase in the gradients of the sum of counts, corresponding to a significant increase in the acoustic signal energy. The failure of the type I sample initiated in the free-wall buckling region (Fig. 17).

**Fig. 18:**
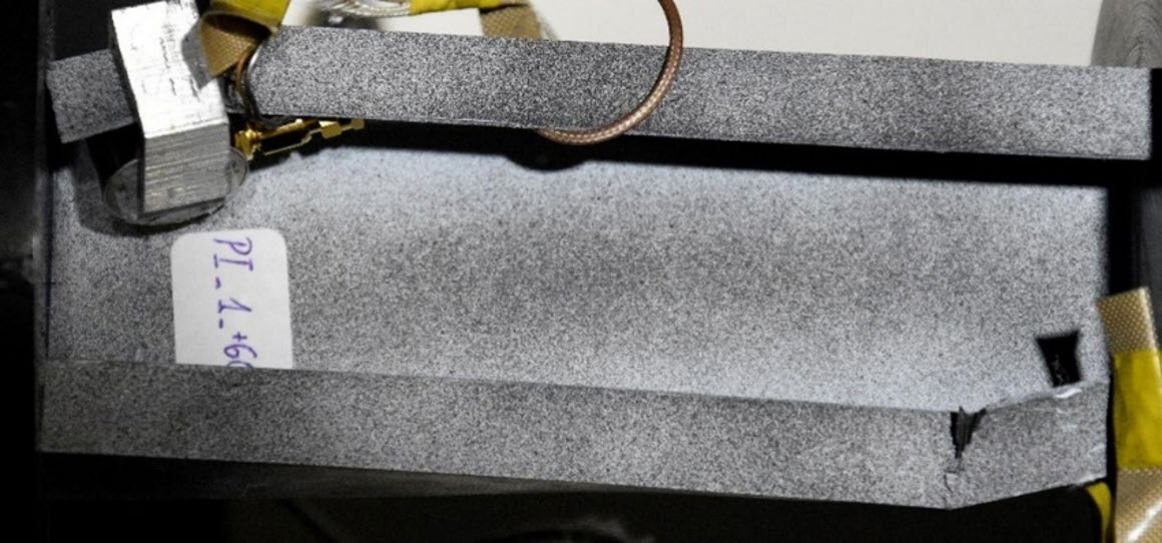
Failure mode of type I sample at 60 °C.

The experiments carried out at + 60 °C showed a similar mechanism of damage processes as in the previous case. This is particularly clear when comparing the representative equilibrium paths obtained for type I samples. They show that the delamination occurs in stages and initiates in different regions of the samples. Each of these stages is accompanied by a rapid increase in the number of acoustic signal counts. In contrast, the increments in acoustic signal energy are relatively small due to a temperature-induced decrease in the matrix stiffness. For both sample types, optical scanning showed significant increments in the deflection of the observed points, corresponding to a decline in the representative equilibrium paths. For type I samples, failure initiation was signalled by a sharp increase in the gradient of the sum of counts, which corresponded to a shortening value of approximately 1.2 mm. The increase in acoustic signal energy can be misleading, as the first clear increase was recorded when the sample shortening was significantly greater. It can therefore be concluded that at elevated temperatures, this characteristic does not have to be relevant due to the reduced stiffness of the matrix. As previously mentioned, the failure of the sample initiates in the free-wall buckling region (Fig. 18).

**Fig. 19:**
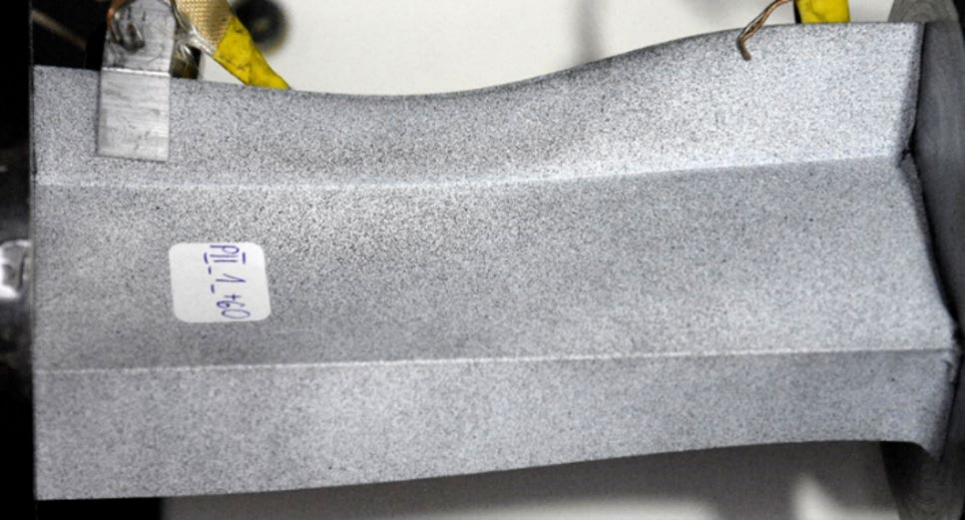
Buckling mode of type II sample at 60 °C.

For type II samples, the first increase in counts occurs at a sample shortening of 1 mm and is accompanied by an increase in acoustic signal energy. This suggests that local delamination occurred within the composite structure, with no apparent effect on the sample’s stiffness. As with previously discussed effects of this type, the phenomenon should be regarded as a signal of impending rapid failure and cannot be ignored in the operation of the structural component. The experiment demonstrated that the effect was accompanied by significant postbuckling, and therefore, this acoustic effect could also be due to the bifurcation phenomenon (Fig. 19). For this case, the composite failure initiated when the sample shortening was approximately 1.4 mm, which is reflected both in the representative equilibrium path and in the sum of counts.

A similar local delamination process was observed for a type I sample in the experiment conducted at + 80 °C. Although the delamination was not accompanied by a sudden increase in the critical buckling nor caused any abrupt change in the gradient of the representative equilibrium path, one could observe a small decline in the path, which was a warning signal. For both sample types, the failure was abrupt and characterized by an increase in the gradient of the sum of counts.

**Fig. 20:**
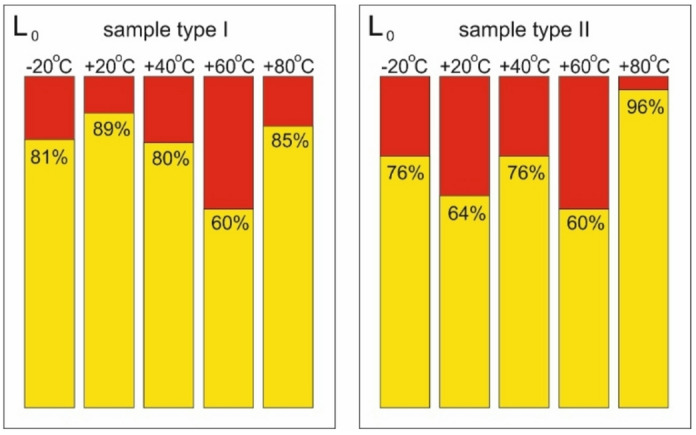
Comparison of L_0_ results.

The diagram below shows the results of the L0 indicator, which describes the ratio between the value of structural shortening corresponding to the first increment in acoustic signal energy and the value of shortening corresponding to the moment of structural failure (Fig. 20).

As shown in the diagram, the results are similar across different temperatures. For type I samples, the representative equilibrium path shows a different failure mechanism at + 60 °C than at other temperatures. This failure process occurred in multiple stages, indicating a gradual weakening of the composite structure through local delamination. It is therefore difficult to determine the moment of failure with certainty, and the adopted value is rather conventional. For type II samples, at + 80 °C, the failure occurred practically simultaneously with the first rapid increase in the acoustic signal energy. However, it is difficult to assess whether the lack of acoustic signal energy increases during earlier loading phases was due to the specific properties of the test sample or a recurring effect.

**Fig. 21:**
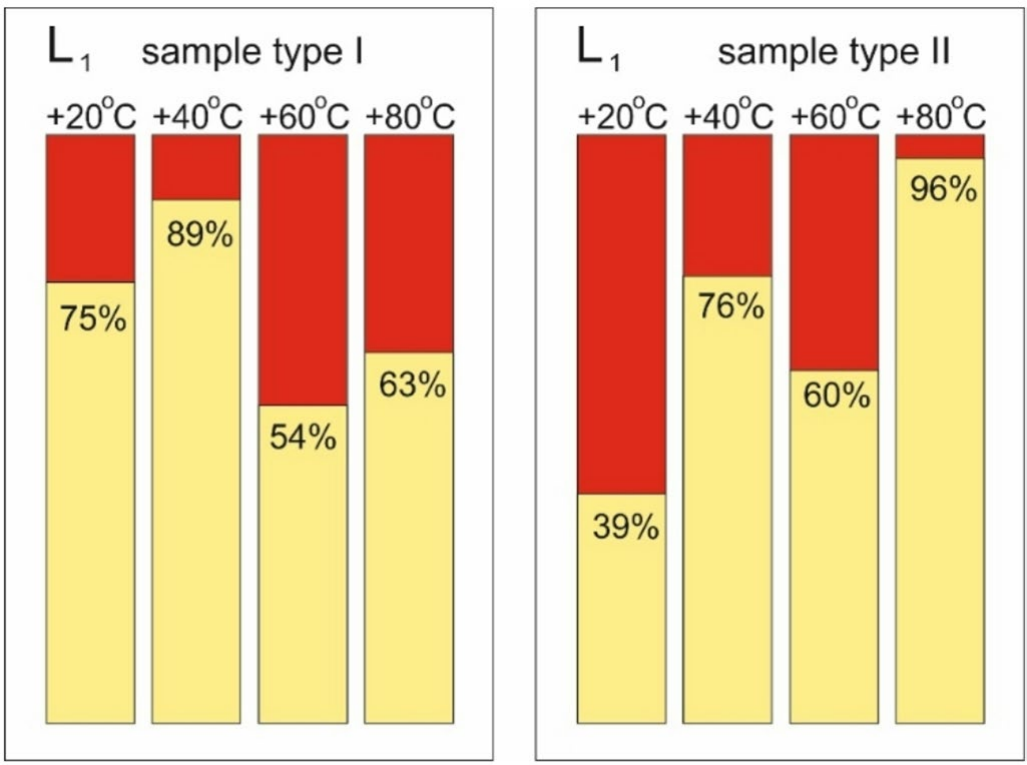
Comparison of L_1_ results.

The next diagram shows (Fig. 21) the results of L1 describing the ratio between the shortening value marking the first significant increase in the count sum gradient to the shortening value corresponding to the moment of failure. In this case, due to the aforementioned acoustic interference, the results obtained at −20 °C are not included.

A comparison of the above results reveals several discrepancies. It is difficult to firmly conclude which of the two coefficients is a real determinant of the loading phase moment when the composite degradation begins. Both seem useful for practical predictive diagnostics under operating conditions. It is thus recommended to follow the rule that the critical warning signal occurs when either indicator has a lower value at a given temperature.

## Conclusions

This paper presents the results of an experimental analysis of the fracture process of thin-walled composite multilayer profiles during compression. Two different cross-section shapes were considered: Ω-type and Z-type. The tests were conducted at room temperature and at elevated and low temperatures, using equilibrium path analysis, digital image correlation, and acoustic emission methods. The results obtained allowed the formulation of several key conclusions, as presented below.

1. It is possible to establish relationships between the results of acoustic emission and deflection and the photographically captured buckling modes and failure mechanisms of the samples. Knowing these relations, acoustic signal emission appears to be an invaluable diagnostic tool for predicting the damage initiation and growth under given operating conditions.
2. For most cases, a sharp change in the gradient of the sum of counts marks the onset of profound structural changes in the composite material. Sometimes, however, this gradient does not change significantly and may be accompanied by single increments in the acoustic signal energy, which indicate the occurrence of the first local delamination and the weakening of the entire structure.
3. It is necessary to establish a simplified procedure for interpreting acoustic signals, one that would make it possible to diagnose critical conditions preceding structural failure. The proposed method uses two indicators. The first one describes the percentage relationship between the sample shortening corresponding to the first sudden increase in acoustic signal energy and the shortening at the moment of failure. The second one defines the percentage relationship between the value of shortening corresponding to the first significant increment in the gradient of the sum of counts, and, as previously, the value of shortening corresponding to the moment of failure.
4. The essence of the proposed method is to prioritise one of the two indicators in such a way as to determine the ‘safe operating range’ for a diagnosed structural condition. In practice, therefore, this condition should be determined based on the indicator that takes a lower value under given operating conditions. The potentially unsafe condition diagnosed thereby would serve as a premise for conducting further diagnostic tests using alternative tools.

It should be emphasised that the effectiveness of the proposed method needs to be validated by further experiments at different temperatures. It is planned that the experiments will be conducted on samples with different composite structures and carbon fabric layer orientations. Furthermore, important papers in the world literature, such as^37–39^, indicate potential future research trends in composite materials.

## Data Availability

The authors confirm that the data supporting the findings of this study are available within the article.
